# Centre-based care is a significant predictor of lower body mass index in early childhood: Longitudinal evidence from Chile

**DOI:** 10.7189/jogh.10.010419

**Published:** 2020-06

**Authors:** Kasim Allel, Marigen Narea, Eduardo A Undurraga

**Affiliations:** 1 Institute for Global Health, University College London, UK; 2 Millennium Nucleus for the Study of the Life Course and Vulnerability (MLIV), Chile; 3 Society and Health Research Centre, Facultad de Humanidades, Universidad Mayor, Santiago, Chile; 4 Centre for Advanced Studies on Educational Justice (CJE), Pontificia Universidad Católica de Chile, Macul, Santiago, Chile; 5 School of Psychology, Pontificia Universidad Católica de Chile, Macul, Santiago, Chile; 6 Escuela de Gobierno, Pontificia Universidad Católica de Chile, Macul, Santiago, Chile

## Abstract

**Background:**

The prevalence of childhood overweight has increased by approximately 50% in the past three decades, becoming a major public health concern worldwide. In Chile, an upper middle-income country, about 38% of children between two and four years of age are overweight, almost double the average in Latin America and the Caribbean. Various environmental and individual factors, and their interactions, affect childhood weight. Emerging evidence suggests childcare may also matter. Because the public provision of centre-based care is growing, childcare may be a useful policy tool to help prevent childhood overweight.

**Methods:**

Using a nationally representative longitudinal survey of ~ 15 000 children in Chile (2010 and 2012), we estimated whether the type of child care (centre-based or maternal) a child attended at age 24 to 36 months was a significant predictor of the child’s sex-and-age-specific body-mass-index (BMI) at age 36-48 months. We restricted our sample to children in full-time maternal care at baseline (12-24 months of age; n = 1273), but tested the robustness of results with the full sample. We compared children in centre-based care and in maternal care using difference-in-difference estimators and propensity score matching, and adjusted our estimates using child, family, and neighborhood characteristics.

**Results:**

Children attending centre-based care had 0.27 SD lower BMI than children in maternal care at follow-up (*P* < 0.05). We found suggestive evidence this association may be modulated by the child’s socioeconomic status and by how frequently the child watched television: we found smaller BMI changes for children at the bottom 80% of socioeconomic status (*P* < 0.05) and also for children who frequently watched television (*P* < 0.10). Our results were robust to various model specifications.

**Conclusions:**

Our findings suggest centre-based care programs, with adequate regulation and enforcement, may be a useful support to help curb the early childhood overweight epidemic, in addition to known effects in labor supply and child development.

Childhood overweight has become a major public health concern in most countries across the globe. The prevalence of childhood overweight, defined as age and sex standardized body mass index (BMI)>two standard deviations (SD) from the World Health Organization (WHO) growth standard median, has increased by approximately 50% in the past thirty years. This increase has not only affected high-income countries, but many middle and low-income countries as well [1-3]. The sometimes called “obesity pandemic” [4,5] is among the most relevant public health debates in the Americas; childhood overweight is particularly high in the United States [6-8] but also in some islands of the Caribbean, and in Mexico, Costa Rica, Uruguay, and Chile [1,9-11]. The prevalence of overweight and obesity in children under 6 years of age in Chile was 34% in 2013 [12]. In 2015, the prevalence of overweight and obesity in children between two and four years of age in Chile (38%) almost doubled the average prevalence of overweight and obesity in Latin America and the Caribbean [13] (**Figure 1**).

**Figure 1:**
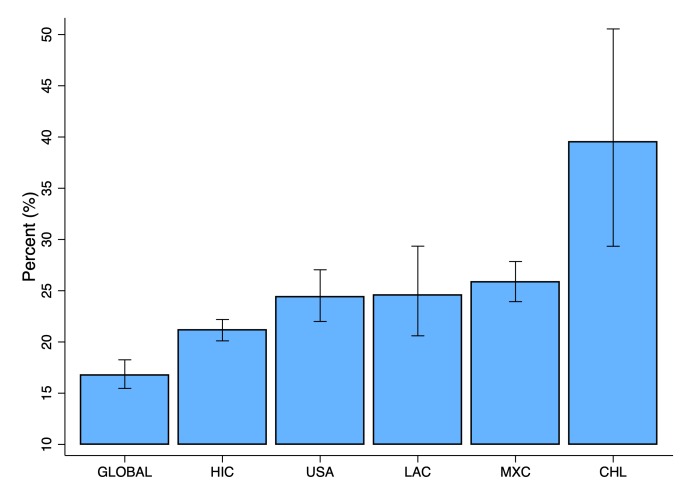
Estimated percentage of overweight or obese children between two and four years of age in selected countries and regions for 2015. Data of prevalence of overweight and obesity in children from GBD Obesity Collaborators [13]. Overweight was defined as BMI-for-age >2 standard deviations from WHO growth standard median. GLB – global rates, HIC – high-income countries, LAC – Latin American and the Caribbean, MXC – Mexico, CHL – Chile.

Several environmental and individual factors, and their interactions, affect childhood weight [4,14]. Environmental factors associated with increases in childhood obesity include changes in food systems such as the increasing availability and accessibility of energy-dense foods [4,15,16], food marketing [4,13], economic development and wealth [16-19], reduced energy expenditure from urbanization and technological change [20]. Obesity is also affected by individual factors, including genetic makeup [21-23], behaviour (eg, physical activity, television viewing, sleep) [24-27], cultural preferences (eg, nutrition, body norms) [28,29], and possibly the microbiome [30]. Complex interactions between environmental and individual factors explain observed variations in obesity rates between population subgroups [4].

A vast amount of research has shown the potential health risks and adverse consequences of childhood overweight and obesity, such as type II diabetes mellitus, respiratory afflictions, and psychosocial stress, and its potentially devastating consequences later in life, including a higher risk of developing cardiovascular diseases, diabetes, musculoskeletal disorders, and some cancers [3,13,31-38]. Early prevention of childhood overweight and obesity has increasingly taken a central stage in the global policy debate [39-45], focusing largely on behavioral changes and lifestyle and environment modifications [15,46-49]. Because young children depend on parental o caregivers’ decisions, changes in the provision of child services, including centre-based care (CBC), may also be important. The term centre-based care (CBC) is often used to refer to a nursery for young children, typically under the age of five [50]. CBC is a relevant policy tool that, in addition to known effects on labor supply and child development [51-53], could potentially help curb the obesity pandemic by, for example, providing adequate nutrition, encouraging physical activity, and promoting parental education [54-58].

Growing evidence suggests childhood overweight could be affected by CBC services, although there is no closure on the magnitude or direction of this association. At least three studies, in Canada, the USA, and The Netherlands [59-61] found that attending CBC in early childhood (1-4 years of age) was associated with a higher prevalence of overweight or obesity. These studies suggested weight gain in CBC may be explained by poor quality or limited regulation of nutritional and physical activity programs. In contrast, at least five studies in the USA have found CBC attendance was negatively associated with child overweight and obesity [62-66]. Broadly, those authors suggest CBC may protect against overweight by promoting a healthier diet and providing health care [62]. Yet other studies have found no association between CBC and childhood overweight [67-74]. Attending CBC could be associated with lower risk of child overweight or obesity through several pathways, including planned nutritional programs and learning environments, healthy eating, regular opportunities for physical activity, regular bedtime and rise time, and reduced screen time (eg, computers, mobile phones, television) [62,63,75,76].

Much of what we know about the relation between CBC and overweight comes from high-income western countries and has focused on type-of-care effects (eg, maternal care, centre-based care). In addition, previous studies have shown heterogeneous sampling designs; most previous studies lack behavioral data (eg, screen time, sleeping), operational data about each specific type of care (eg, main source of funding,), and hours spent in CBC. Most previous estimates of the relation between CBC and overweight are associative, and could thus be biased by omitted confounders, such as access to child care, demographic heterogeneity (eg, race, ethnicity), and/or CBC characteristics [77].

We estimated whether the type of child care (centre-based or maternal) at age 24 to 36 months was a significant predictor of a child’s (i) sex-and-age-specific body-mass-index (BMI) and (ii) the probability of being overweight (BMI>two standard deviations, SD), at age 36-48 months. Using a nationally representative longitudinal survey of ~ 15 000 children in Chile (2010 and 2012), we compared children in centre-based care (CBC) and in maternal care using difference-in-difference estimators and propensity score matching (PSM) to reduce omitted variable biases.

We think Chile is an apt location for this study for at least three reasons. First, most evidence on the association between CBC and childhood overweight comes from industrialized countries. Second, Chile has the highest rates of childhood overweight and obesity in South America [12,78]. Third, the Chilean government provides subsidized CBC for children from the first three quintiles of household income distribution since 2006 [79,80], with government-regulated dietary intake and infrastructure. Child enrolment in publicly funded CBC has thus increased substantially during the past decade, with 67% of children <5 years of age enrolled in CBC in 2017 [81]. Public CBC programs include three meals for children enrolled full time (breakfast, lunch, and an afternoon meal), providing about 60% of daily calories required (about 800 calories) [82]. Despite recent efforts to improve social programs for child development, Chile is among several middle income countries where childhood overweight is a major public health concern; our results provide external validity to previous findings with comparable CBC coverage and childhood overweight to the US and Canada [83,84]. To our knowledge, this is the first study to explore CBC as a plausible policy intervention that could help curb the childhood obesity epidemic in Latin America.

## METHODS

### Data sample

**Figure 2:**
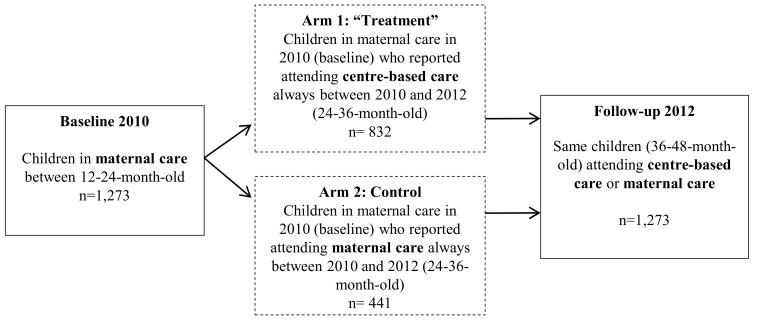
Definition of the study sample.

We used data from the Chilean Early-Childhood Longitudinal Survey (ELPI, 2010 and 2012) [85], a nationally representative data set of about 15 000 children between six months and five years of age at the time of the 2010 survey (ie, born between January 1, 2006 and August 31, 2009). ELPI [85] includes household-level sociodemographic, economic, and educational data, and several child-development measures, including anthropometrics. Using a baseline (2010) sample of children in maternal care between 12 and 24 months of age, we compared indicators of sex- and age-specific BMI for children in CBC and in full-time maternal care at follow up (2012; n = 1273; **Figure 2**).

### Health outcomes

Data on children’s weight and height were collected in each survey round by trained research staff using a portable stadiometer and scale, during a household visit. We computed BMI (kg/m^2^) and defined overweight as having >2 SD from the age-sex standardized growth median BMI of a cohort of well-nourished international population [86,87].

### Variables

Attendance to CBC was asked retrospectively for children of 0-24 and 24-36 months of age (“Did your child attend CBC between the age of 24 to 36 months?”; 1 = child attended CBC, 0 = otherwise). Parents were also asked about the child’s main caregiver at ages 0 through 24 months. We restricted the sample to children in maternal care at baseline (2010). Household and family background are associated with childhood overweight [88,89], particularly parental overweight [90]. We used mother characteristics as a proxy for parental characteristics since all households in our sample had data on mother’s characteristics. **Table 1** shows variable definitions.

**Table 1:** Definition of variables used in the main analysis*

Variable name	Definition
**Dependent variables (outcomes)**	
BMI and Delta-BMI	BMI is defined as child weight divided by body height squared kg/m^2^). Delta-BMI was calculated as child’s BMI in 2012 – child’s BMI in 2010
Overweight	1 = BMI-for-age >2 SDs from WHO growth standard median; 0 = otherwise
**Child characteristics**	
Age†	Age of the child at the time of the survey (in months)
Female	Child’s gender. 1 = girl, 0 = boy
Television	Number of hours (range) that the child spends watching television on a typical day. 0 = child does not watch television, 1 = less than 1 h per day, 2 = 1-2 h per day, 3 = 2-3 h per day, 4 = 3-4 h per day, 5 = 4-5 h per day, 6 = more than 5 h per day
Premature	1 = the child was born prematurely (<36 gestational weeks); 0 = otherwise
Video-games	Number of hours the child spends using a cell-phone in a typical day (0-6 h)
Sleep	Total number of hours that the child sleeps during a typical day
Caesarean	1 = child born by caesarean section; 0 = vaginal delivery
Illness	Number of illnesses that the child has experienced since birth. Ranges from 0 to 12, assigns one point for each of the following illnesses: respiratory, stomach, kidney, growth, visual, listening, skin, learning, mental health, traumatology, dental, neurological, and motor problems
Older sibling	1 = child has an older sibling; 0 = otherwise
BMI at birth	1 = child had BMI<25^th^ percentile at birth; 0 = otherwise
**Maternal characteristics**	
Age†	Mother’s age at the time of survey (years)
Ethnicity	1 = mother speaks at least one indigenous language, 0 = otherwise
BMI	Weight divided by body height squared (kg/m^2^)
Overweight status	1 = BMI>2 SD from growth standard median; 0 = otherwise
Married	1 = mother was married at the time of the survey; 0 = otherwise
Worked before birth	1 = mother was working before child’s birth; 0 = otherwise
Smoke	1 = mother smoked during pregnancy; 0 = otherwise
Depression	1 = mother has been diagnosed with depression (self-reported); 0 = otherwise
Chronic disease	Number of chronic diseases that have affected the child’s mother. Ranges from 0-22, with 1 for each of the following diseases: lung, stomach, kidney, growth, listening, visual, skin, learning, traumatology, cancer, diabetes, hypertension, heart, obesity, depression, anxiety, bipolarity, schizophrenia, autism, hyperactivity, alcoholism, and drug problems
Cigarettes	Number of monthly cigarettes smoked during the child’s first 6 months of age
**Household characteristics**	
SES	Index of SES based on five variables: parental occupation from low to high skills (range of 9 values), parental years of schooling, household income per person [91]. Index ranges from -2.5 to 2.5; higher scores indicate higher SES
Region	Categorical variable indicating region of residence in Chile (0-15)
HOME score	Abbreviated version of the Home Observation for Measurement of the Environment (HOME). The score measures quality of social, emotional, and cognitive support for children at home (ranges from 0-22, with higher numbers indicating better environment) [92].
**Other covariates**‡	
SES status (40%, 60%, 80%)	Categorical variable indicating whether the children belonged to the 40%, 60%, or 80% most vulnerable children by SES (1 = child was among the X% more vulnerable; 0 = otherwise)
Part-time	1 = child attended a CBC during 4 or less hours in a typical day; 0 = otherwise
Full-time	1 = child attended a CBC full time (8 h per day); 0 = otherwise
Private	1 = child attended private CBC program; 0 = otherwise
Public	1 = child attended public CBC program; 0 = otherwise

BMI – body mass index, CBC – centre-based care, SES – socioeconomic status, SD – standard deviation.

*There was 18% attrition between baseline and follow-up.

†Age squared was also used in the regressions for the main analysis.

‡Interacted with CBC attendance to present joint associations in separate models.

### Statistical analysis

#### Main models

We used three main model specifications to understand the association between type of childcare and short-term changes in child’s BMI. We reduced bias from potentially confounding variables that predict selection into CBC (“treatment”), using propensity score matching (PSM) to generate two comparable groups. PSM uses a logistic regression to predict participation in CBC. Based on observable characteristics at baseline (covariates shown in **Table 1**), children in the sample receive a propensity score and are then matched to create statistically comparable groups of treated (CBC) and non-treated (maternal care) children [93] (see supplementary material for further details).

In Model 1 we used a weighted difference-in-differences Ordinary Least Squares [47] regression with individual time-unvarying covariates and robust standard errors (Equation 1):

(*BMI_i,t_ – BMI_i,t–_*_1_) = *β*_0_ + *β*_1_ (*CBC_i,t_*) + Σ*β_k_X_k,i,t-_*_1_ + *ϵ*_i,t_  (Eq 1)

Where the subscripts (*i*) stand for child, (*k*) for the covariates included, and (*t*) for time, with *t* = follow-up survey and *t – 1* = baseline. BMI denotes age- and sex-specific body-mass-index and CBC indicates whether the child attended CBC (CBC = 1) or maternal care (CBC = 0). Because we used PSM, *β_1_* captures the average treatment effect of attending CBC compared to maternal care, as individuals were “treated” in 2012 (average treatment effect on the treated). *X_k_* is a vector of explanatory covariates at baseline (**Table 1**). ϵ is an error term.

Model 2 was based in Equation 1, but allowed individual’s characteristics to vary in time (*X_k,i,2012_* – *X_k,i,2010_*), using time-varying covariates with PSM (Equation 2):

(*BMI_i,2012_ – BMI_i,2010_*) = *β*_0_ + *β*_1_ (*CBC_i,2012_*) + Σ*β_k_ (X_k,i,2012_* – *X_k,i,2010_*) +  (*ϵ*_i,2012_)  (Eq 2)

In Model 3, we used short-term General Linear Squares with fixed-effects and PSM added as weights (Equation 3). We used BMI as dependent variable and kept the time-varying covariates from Model 2. Model 3 allowed us to compare the robustness of estimates in Model 1 and Model 2, and to estimate individual’s fixed effects between waves given by *γ_i_* (time-invariant differences between individuals). *β_1_* in this model shows the linear change in BMI from attending CBC:

*BMI_i,j_* = *β*_0_ + *β*_1_ (*CBC_i,2012_*) + Σ*β_k_X_i,j_* + *ϵ*_i,j_  + *γ*_i_ (Eq 3)

#### Additional analysis

We provided two additional analyses. First, we tested the robustness of the association between attending CBC and BMI, by examining a simpler model without covariates, and also by comparing CBC with any other type of care in the survey. Second, we explored the mechanisms through which the type of CBC could affect BMI. In Model 4 we expanded Model 1 and Model 2 by adding plausible moderators of the association between CBC and child weight (SES group, frequency of watching television, part-time or full-time attendance to CBC, and whether CBC was public or private), and an interaction term between these moderators and CBC.

All analyses were done using STATA 15.1 (StataCorp, College Station, TX, USA) and/or RStudio Version 1.1.383. Inc. Boston MA, EEUU.

## RESULTS

### Descriptive statistics

**Table 2** shows descriptive statistics of the sample at baseline (2010). About 21% of children between 12 and 24 months of age were overweight or obese at baseline (2010) (Figure S1 in the **Online Supplementary Document**). Approximately one third of the sample (35%) attended CBC programs between 24 and 36 months of age. Compared to children in maternal care, children enrolled in CBC watched less television, were more frequently ill, and slept fewer hours (*P* < 0.01).

**Table 2:** Descriptive statistics of the sample at baseline (2010), children 1-2 years of age in maternal care*

	Type of care at 2-4 years		T-test	Total
	**Maternal care**	**Centre-based care**		
**Child's characteristics**	**Mean ± SD**	**Mean ± SD**	***P*-value**	**Mean ± SD**
BMI	17.66 ± 1.96	17.87 ± .92	0.09	17.73 ± 1.95
Overweight or obesity (%)	18.19 ± 38.6	20.18 ± 40.18	0.39	18.88 ± 39.15
Age	19.43 ± 2.94	18.84 ± 2.66	0.04	19.18 ± 2.85
Female (%)	50 ± 50.3	47.23 ± 49.98	0.43	49.04 ± 50.01
Television†	2.58 ± 1.56	2.32 ± 1.38	<0.001	2.48 ± 1.51
Premature child (%)	7.51 ± 26.37	8.2 ± 27.47	0.59	7.75 ± 26.75
Video-games†	0.67 ± 1.01	0.67 ± 0.96	0.97	0.67 ± 0.99
Sleep†	10.92 ± 1.25	10.48 ± 1.09	<0.001	10.77 ± 1.21
Caesarean (%)	41.48 ± 49.3	41.56 ± 49.34	0.79	41.51 ± 49.29
Illness	0.75 ± 0.82	0.92 ± 0.90	<0.001	0.81 ± 0.85
Older sibling (%)	63.5 ± 48.17	52.77 ± 49.98	<0.001	59.79 ± 49.05
BMI at birth	13.72 ± 1.5	13.68 ± 1.51	0.64	13.71 ± 1.50
**Mother's characteristics:**				
Age	28.84 ± 7.05	27.21 ± 7.02	<0.001	28.25 ± 7.09
Ethnicity (%)	8.49 ± 27.89	6.92 ± 25.41	0.35	7.95 ± 27.06
Overweight status (%)	64.62 ± 47.84	57.56 ± 49.48	0.05	62.17 ± 48.51
Married (%)	78.87 ± 40.84	72.51 ± 44.7	0.01	76.67 ± 42.31
Worked before birth (%)	19.15 ± 39.37	21.95 ± 41.44	0.23	20.12 ± 40.11
Smokes (%)	8.94 ± 28.55	11.56 ± 32	0.11	9.85 ± 29.81
Depression (%)	10.45 ± 30.61	14.63 ± 35.38	0.04	11.9 ± 32.4
Chronic disease	0.83 ± 1.32	1.01 ± 1.55	0.03	0.89 ± 1.41
Cigarettes	6.12 ± 3886	6.65 ± 29.45	0.78	6.35 ± 35.87
**Household characteristics:**				
SES	-0.04 ± 0.66	0.08 ± 0.70	0.01	0.00 ± 0.68
Region	8.92 ± 3.77	8.75 ± 4	0.45	8.88 ± 3.85
HOME score	14.92 ± 3.19	15.16 ± 3.14	0.20	14.99 ± 3.17
Number of participants	832	441		1273

BMI – body mass index, SD – standard deviation, SES – socioeconomic status, HOME – The Home Observation for Measurement of the Environment

*See Table S2 in the **Online Supplementary Document** for descriptive statistics following propensity score matching.

†Behavioral covariates added to the analysis in 2012.

### Propensity score matching

Based on observable characteristics at baseline, PSM showed the predicted probability of attending CBC was comparable for children who remained in maternal care (untreated) and children who attended CBC (treated) (Tables S1 and S2, and Figure S2 in the **Online Supplementary Document**). The number of hours the child spent watching television and sleeping were negatively associated with the likelihood that the child attended CBC (β_TV_ = 0.15, *P* < 0.01; β_sleep_ = -0.32, *P* < 0.01). In contrast, SES and the number of illnesses the child had experienced since birth were positively associated with the likelihood of attending CBC (β_SES_ = 0.22, *P* < 0.05; β_Illnesses_ = 0.18, *P* < 0.05).

### Main results

**Table 3** shows the main results from the regression analysis: attending CBC was associated with lower BMI across models. **Table 3**, Model 1, suggests that compared with children who were always in maternal care, children who attended CBC between 2010 and 2012 saw an average decrease of 0.27 SD of their BMI (β_CBC_ = -0.27, *P* = 0.03). Results in Model 2, using a full difference-in-differences model accounting for the variability in child’s characteristics in time, are consistent with Model 1. Being enrolled in CBC in 2012 was associated with a .26 SD reduction in BMI compared to maternal care (β_CBC_ = -0.26, *P* = 0.05). Last, Model 3, shows comparable results when using fixed effects to isolate the individual’s unvarying and heterogeneous characteristics (β_CBC_ = -0.25, *P* = 0.07).

**Table 3:** Association between the type of care and changes in BMI-for-age for children between two and four years of age

	Model 1		Model 2		Model 3	
	**OLS DID with PSM**		**OLS DID with PSM**		**FE with PSM**	
**Main independent outcomes**	**β (SE)**	*P*-value*	**β (SE)**	*P*-value*	**β (SE)**	*P*-value*
**Child characteristics:**						
Centre-based care	-0.27 (0.13)	0.03	-0.26 (0.14)	0.05	-0.25 (0.14)	0.07
Age	0.26 (0.24)	0.28	-0.10 (0.05)	0.04	-0.12 (0.03)	<0.001
Age squared	0.00 (0.01)	0.46	0.00 (0.00)	<0.001	0.00 (0.00)	<0.001
Female	0.200 (0.14)	0.13	-	-	-	-
Television	0.01 (0.05)	0.94	-	-	-	-
Premature	0.12 (0.23)	0.60	-	-	-	-
Video-games	-0.13 (0.07)	0.03	-	-	-	-
Sleep	-0.04 (0.06)	0.54	-	-	-	-
Caesarean	0.03 (0.14)	0.78	-	-	-	-
Illness†	0.05 (0.08)	0.52	0.00 (0.06)		-0.01 (0.06)	0.92
Older Sibling	-0.02 (0.17)	0.86	-	-	-	-
BMI at birth	0.19 (0.14)	0.17	-	-	-	-
**Mother characteristics:**						
Age	0.13 (0.07)	0.06	-0.03 (0.17)	0.86	-0.01 (0.17)	0.94
Age squared	-0.00 (0.00)	0.03	0.00 (0.00)	0.35	0.00 (0.00)	0.21
Ethnicity	-0.03 (0.33)	0.96	-	-	-	-
BMI	0.05 (0.01)	<0.001	0.03 (0.02)	0.10	0.04 (0.02)	0.05
Married‡	-0.08 (0.16)	0.63	0.00 (0.14)	0.99	-0.01 (0.15)	0.95
Worked before birth	0.00 (0.17)	0.97	-	-	-	-
Smokes	0.21 (0.23)	0.35	-	-	-	-
Depression	0.02 (0.20)	0.89	-	-	-	-
Chronic disease†	0.00 (0.06)	0.94	0.00 (0.14)	0.99	0.02 (0.14)	0.87
Cigarettes	0.00 (0.00)	0.28	-	-	-	-
**Household characteristics:**						
SES	0.20 (0.10)	0.03	0.03 (0.15)	0.83	-0.01 (0.15)	0.96
Region	0.01 (0.02)	0.58	-	-	-	-
Constant	-6.51 (2.45)	0.01	-0.47 (0.93)	0.61	21.19 (3.65)	<0.001
Number of individuals	1273		1268		1268	

BMI – body mass index, DID – difference in differences, OLS – ordinary least squares, FE –fixed effects model, PSM – propensity score matching, SES – socioeconomic status.

*Two tailed tests were employed for *P* values estimation.

†Illness and chronic disease were replaced by dichotomous variables expressing a change between panel waves in the Full DID and FE models, ie, the appearance of a chronic condition or child illness.

‡For the full DID and FE models, married reflects a change in marital status, ie, becoming unmarried between panel waves.

To be sure our results did not hinge on model specification, we further examined the association between CBC and BMI (i) excluding all covariates, with and without propensity score matching (Table S3 in the **Online Supplementary Document**), and (ii) comparing CBC with any other type of care (ie, maternal care, grandparent care, care by acquaintances/relatives) using a larger sample (Table S4 in the **Online Supplementary Document**). Overall, these results show that the negative association between CBC and BMI is robust.

### Additional results

To understand the mechanisms that may explain our main findings, we examined the interaction between CBC and (i) SES, (ii) television, (iii) full or part-time attendance to CBC, and (iv) main source of funding (private or public). **Table 4** shows several interesting findings. The association between CBC and BMI remained negative, large in magnitude, and statistically significant in most models. **Table 4**, Panel A.1, suggests the magnitude of the association between attending CBC and BMI was smaller for children at the bottom 80% of SES (β_CBC_ + β_80%SES×CBC_ = -0.71 + 0.55 = -0.16, F-joint test = 3.84; *P* = 0.02), than for children at the top 20% of SES (β_CBC_ = -0.71; *P* = 0.003). We examined the interaction between CBC and children at the second and third quintiles of SES, but found no significant results (Table S5 in the **Online Supplementary Document**). We also found suggestive evidence that watching television diminished the magnitude of the negative association between CBC and BMI (β_TV×CBC_ = 0.15; *P* = 0.09). The results in **Table 4**, Panel A.2, suggest the decrease in BMI from attending CBC may even disappear for children watching more than 4 hours of television per day (β_CBC_ + β_TV×CBC_ = 4* *×* * [0.15]-0.64 = -0.04, F-joint test = 3.65; *P* = 0.03). Attending CBC full-time was significantly associated with lower BMI (β_FT_ = -0.46, *P* < 0.001), but the association was not significant for children attending CBC only part-time. Last, we found only suggestive evidence public funding could also mediate the association between CBC and BMI (β_public_ = -0.24, *P* = 0.08).

**Table 4:** Plausible mechanisms to explain the association between attending centre-based care and BMI-for-age in children between two and four years of age (Model 4)

	DID w/PSM (N = 1273)		Full DID with PSM (N = 1268)	
**Main outcome in the regression**	**β (SE)**	*P*-value*	**β (SE)**	*P*-value*
**A. Lifestyle moderators**				
**A.1: SES (80% more vulnerable)**†				
Centre based care	-0.71(0.26)	<0.001	-0.74 (0.26)	<0.001
SES<80% × Centre based care	0.55 (0.31)	0.06	0.60 (0.31)	0.05
SES<80%	-0.45 (0.21)	0.03	-0.46 (0.21)	0.03
Constant	-6.23 (2.47)	<0.001	-0.18 (0.93)	0.84
**A.2: Television:**				
Centre based care	-0.64 (0.25)	0.01	-0.68 (0.26)	<0.001
Television × Centre-based care	0.15 (0.09)	0.09	0.17 (0.09)	0.07
Television	-0.06 (0.05)	0.22	-0.09 (0.05)	0.09
Constant	-6.24 (2.43)	0.01	-0.19 (0.94)	0.84
**B. Centre-based care moderators**				
**B.1: Full and part-time assistance**				
Full-time	-0.46 (0.17)	<0.001	0.46 (0.16)	<0.001
Part-time	-0.06 (0.17)	0.06	-0.05 (0.18)	0.06
Constant	-6.10 (2.46)	0.01	-0.49 (0.93)	0.60
**B.2: Public and private funding**				
Private	-0.36 (0.25)	0.15	-0.25 (0.23)	0.10
Public	-0.24 (0.15)	0.08	-0.26 (0.15)	0.08
Constant	-6.58 (2.43)	<0.001	-0.45 (0.94)	0.60

DID – difference in differences, PSM – propensity score matching, SES – socio-economic status.

*Two tailed tests were employed for *P*-values estimatio. We used the same covariates as shown in **Table 3**.

†Results including children at in the lower second and third quintiles of socioeconomic status (lower 40% and 60% respectively) are shown in Table S5 in the **Online Supplementary Document**.

Changes in BMI do not necessarily imply a change in weight status category (ie, underweight, normal weight, overweight, or obese). Weight status categories matter as a health metric, for example, to understand trends or measure health progress. We used a logistic regression to examine the association between attending CBC and the probability of a child being classified as overweight in 2012 (Table S6 in the **Online Supplementary Document**). We found no statistically significant association between CBC and overweight status. But being a boy (β_Female_ = -0.33; odds ratio (OR) = 0.72; *P* < 0.05), caesarean-born (β_cesarean_ = 0.34; OR = 1.41; *P* < 0.05), and having a mother with overweight (β_Motherweight_ = 0.37; OR = 1.45; *P* < 0.05) were associated with a greater probability of child overweight (Table S6 in the **Online Supplementary Document**).

**Figure 3:**
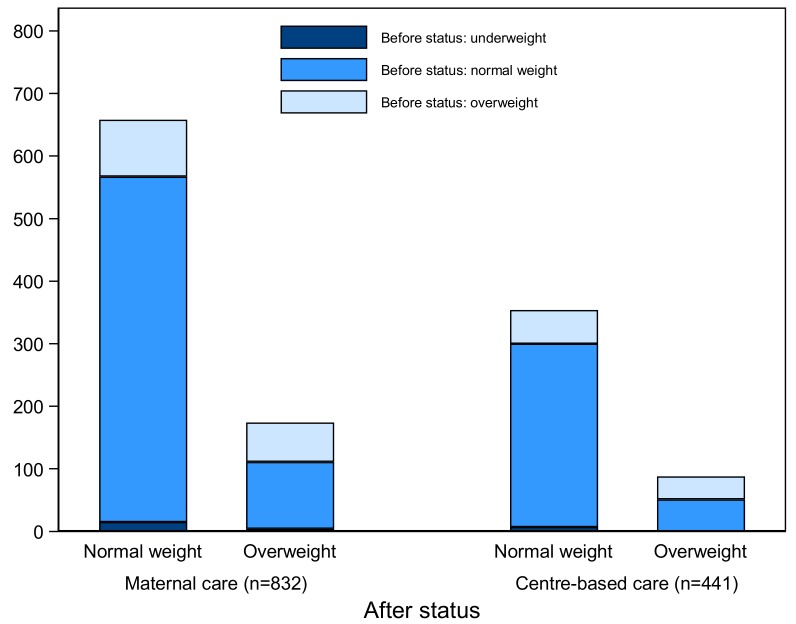
Changes in weight category between 2010 (before status) and 2012 (after status) for children at the age of 36-48 months. Underweight category is not reported in 2012, as they represented less than 1% of the sample. Overweight rate was 22% in 2010 for children in maternal care, while 26% for children in centre-based care, however, they changed to 26% and 24% in 2012.

We last examined the relation between type of care and changes in BMI, focusing on transitions in weight status category. **Figure 3** shows the change in weight status category between 2010 (children were 12-24 months old) and 2012 (children were 36-48 months old), by type of childcare (CBC and maternal). Overall, **Figure 3** shows there were more overweight children in maternal care than in CBC. Between 2010 and 2012, the proportion of overweight children increased from 22% to 26% ( ~ 4%) for children in maternal care and decreased from 26% to 24% ( ~ -2%) for children in CBC.

## DISCUSSION

Based on a difference-in-difference analysis with PSM, our main results suggest that (i) attending CBC, compared to maternal care, was associated with lower BMI in early childhood, and (ii) this association was larger for children attending full-time care, and at the top 20% of SES, and smaller for children who watched television. (iii) Being a boy, caesarean born, and having a mother with overweight, increased the probability of child overweight. Potential policy implications include preventing childhood overweight by favoring access to CBC programs during early childhood.

First, our results are consistent with studies from high-income countries showing CBC programs, compared to maternal care, may help prevent children’s gains in BMI and thus reduce their risk of becoming overweight [62-66]. One probable explanation for these findings relates to existence of a regulated, structured feeding pattern for children attending CBC (ie, three meals per day with balanced nutrition) [82], compared to more free feeding practices under maternal care [94,95]. This hypothesis is consistent with the fact that attending CBC full-time was significantly associated with lower BMI (*P* < 0.001), but the association was not significant for children attending CBC only part-time. The finding that CBC programs, compared to maternal care, may help prevent children’s gains in BMI could have important policy implications, in the context of increasing childhood obesity. While our study has a stronger claim to causality than previous studies, and we have partially addressed common biases in previous literature, the “true” effects of CBC still need to be confirmed using a prospective study design that allows controlling for unobservable variables.

Second, we found suggestive evidence that the association between BMI and CBC may be modulated by the child’s SES status and by how frequently the child watched television. Specifically, we found larger BMI changes for children at the top 20% of SES compared to children at the bottom 20% of SES. This could be explained by differences in the intensity of exercise – that in turn depends on the centres’ infrastructure – and differences in the quality of food in centres attended by children of different SES [96], particularly for children in the top quintile of income distribution.

We also found smaller changes in BMI for children who more frequently watched television. Some studies have found attending CBC may reduce the total time a child watches television [62,63,75]. Full-time enrolment at CBC was also a significant mediator of the association between CBC and BMI; a plausible pathway is through active-learning and improved nutrition at CBC [62,63,75]. We found only suggestive evidence that public funding could mediate the association between CBC and BMI, which would be consistent with the mediating effect of SES. Publicly funded CBC programs, such as Head Start in the USA, have also found lower risk of child overweight [62,63,75].

Third, we examined factors that could explain changes in children’s weight status. Being born by caesarean section, being a boy, and having a mother classified as overweight, were all risk factors for childhood overweight, consistent with previous findings in the literature [39-44,97].

It is important to highlight these results were obtained using data from Chile, where the government provides subsidized CBC for children at the bottom 60% of the income distribution. Dietary intake (three meals) and childcare infrastructure are regulated and adequate quality standards enforced [79,80]. Regulation and enforcement of minimum quality standards makes the CBC “treatment” reasonably comparable in Chile. But results should be interpreted with caution as they may not necessarily apply to other low and middle-income countries, where there may be more variability in regulation and feeding standards.

### Limitations

This study has at least four limitations. First, treatment and control groups were not randomly assigned, which would have allowed us to identify the “true” effect of CBC on BMI. It is possible that children attending CBC were systematically different from children who remained in maternal care. We minimized the risk of systematic bias by restricting our sample to children in maternal care at baseline and using PSM to generate a statistically comparable sample of children in CBC and maternal care based on observable characteristics at baseline. The results from PSM (supplementary material) confirmed that the predicted probability of attending CBC was comparable for children who attended CBC and those who remained in maternal care for all relevant observable characteristics. Second, ELPI survey lacks measures related to specific dietary intake, a major driver of overweight in children. We tried to address this limitation by including lifestyle variables correlated with dietary intake [98,99], and mother’s weight status category as a proxy for household dietary intake. Third, we did not have any measure of CBC quality. We included a limited amount of CBC service (attendance time and type of funding), but did not have relevant information such as teacher-pupil ratio, curriculum, class size, and other proxies of CBC quality. Fourth, we could not assess children’s physical activity, due to lack of data, but included some behavioral and household characteristics partially addressing this limitation. Our findings may need to be confirmed using a study design with a more robust claim to causality, including a more exhaustive range of factors associated with childhood obesity. Replicating this study in other middle- and low-income countries could provide additional external validity to our findings.

## CONCLUSIONS

Our results suggest CBC programs, with adequate regulation and enforcement, could help curb the obesity epidemic in early childhood, in addition to known benefits of CBC [52,53]. The benefits from these programs could extend for many years, including avoiding health complications later in life. CBC programs, particularly in the context of middle-income countries, may help control BMI increases through improved nutrition, physical activities, and other lifestyle-related measures. If adequate care is provided, there is probably a substitution effect between potentially harmful activities that consume the child’s time (eg, watching television) and CBC attendance. A recent review [100] examined nutritional and physical activity regulations for CBC in the USA that could help reduce childhood overweight and obesity. The article highlighted some of the challenges of regulation, and the importance of enforcement to secure quality. Here we show CBC is potentially a good option to curb childhood overweight in middle income countries.

## Additional material

Online Supplementary Document
