# Supplementary material for: Centre-based care is a significant predictor of lower body mass index in early childhood: Longitudinal evidence from Chile
Source: J Glob Health. 2020 Apr 15;10(1):010419. doi: 10.7189/jogh.10.010419 (PMC7182360; doi:10.7189/jogh.10.010419)
Supplement: Online Supplementary Document [file jogh-10-010419-s001.pdf]

**Supplementary online material**

# Center-based care is a significant predictor of lower body mass index in early childhood: Longitudinal evidence from Chile

Kasim Allel[a,b,c], Marigen Narea[d,e], Eduardo A. Undurraga[b,f]

[a] Institute for Global Health, University College London, United Kingdom.

[b] Millennium Nucleus for the Study of the Life Course and Vulnerability (MLIV), Chile.

[c] Society and Health Research Center, Facultad de Humanidades, Universidad Mayor, Santiago, Chile.

[d] Center for Advanced Studies on Educational Justice (CJE), Pontificia Universidad Católica de Chile, Av. Vicuña Mackenna 4860, Macul, Santiago, Región Metropolitana 7820436, Chile.

[e] School of Psychology, Pontificia Universidad Catolica de Chile Av. Vicuña Mackenna 4860, Macul, Santiago, Región Metropolitana 7820436, Chile.

[f] Escuela de Gobierno, Pontificia Universidad Católica de Chile, Av. Vicuña Mackenna 4860, Macul, Santiago, Región Metropolitana 7820436, Chile.

## Contents

1. Methods: model specification ........ 2
   i) Model specification A: Propensity score matching estimation. ........ 2
   ii) Model specification B: Logistic regression........ 2
2. Supplementary results ........ 3
   2.1 Descriptive results: body mass index, children 13-24 months old in 2010........ 3
   2.2 Propensity score matching estimation and balance of the sample after matching ........ 4
   2.3 Robustness checks: raw results of the association between center-based care and BMI........ 7
   2.4 Additional results: mechanisms that could explain main findings........ 11

## 1. Methods: model specification

### i) Model specification A: Propensity score matching estimation.

*Propensity score matching estimation: Logit model to determine the propensity score*

$$1.\text{- ATT: } (\delta_1) = E(\text{BMI}_{1i} - \text{BMI}_{0i}) |\ CBC_i = 1$$

$$2.\text{- } P(CBC = 1|X_i, \ldots, X_n) = \frac{e^{(\alpha+\beta_k X_{ij})}}{1+ e^{(\alpha+\beta_k X_{ij})}} \ ;$$

Then the scores obtained were used as weights as the following:

$$\text{Treated case weight} = \frac{1}{\text{PS}}$$

$$\text{Untreated case weight} = \frac{1}{1-\text{PS}}$$

**Notes:** CBC: center-based care, PS: propensity score, ATT: average treatment effect on the treated

### ii) Model specification B: Logistic regression.

We used a logistic regression (Eq. 4) to estimate the probability of a child being overweight at follow-up (dichotomous variable, child was overweight=1, otherwise=0). $\beta_k$ shows the change associated to a unit increase in the covariate "*k*" on the log odds of the probability of being overweight, or an $e^{\beta k}$ change in the odds of the probability of being overweight:

$$\ln\left[\frac{\Pr(\,Overweight_{\,i} = 1 \mid X_i)}{1-\Pr(\,Overweight_{\,i} = 1 \mid X_i)}\right] = \alpha_0 + \beta_k X_{ki} + \mu_0 \qquad \text{(Eq. 4)}$$

**Notes: "**Pr" stands for probability, and "ln" for natural logarithm.

## 2. Supplementary results

### 2.1 Descriptive results: body mass index, children 13-24 months old in 2010

**Figure S1**. Body-mass index (BMI) by density and categories across children's age in 2010.

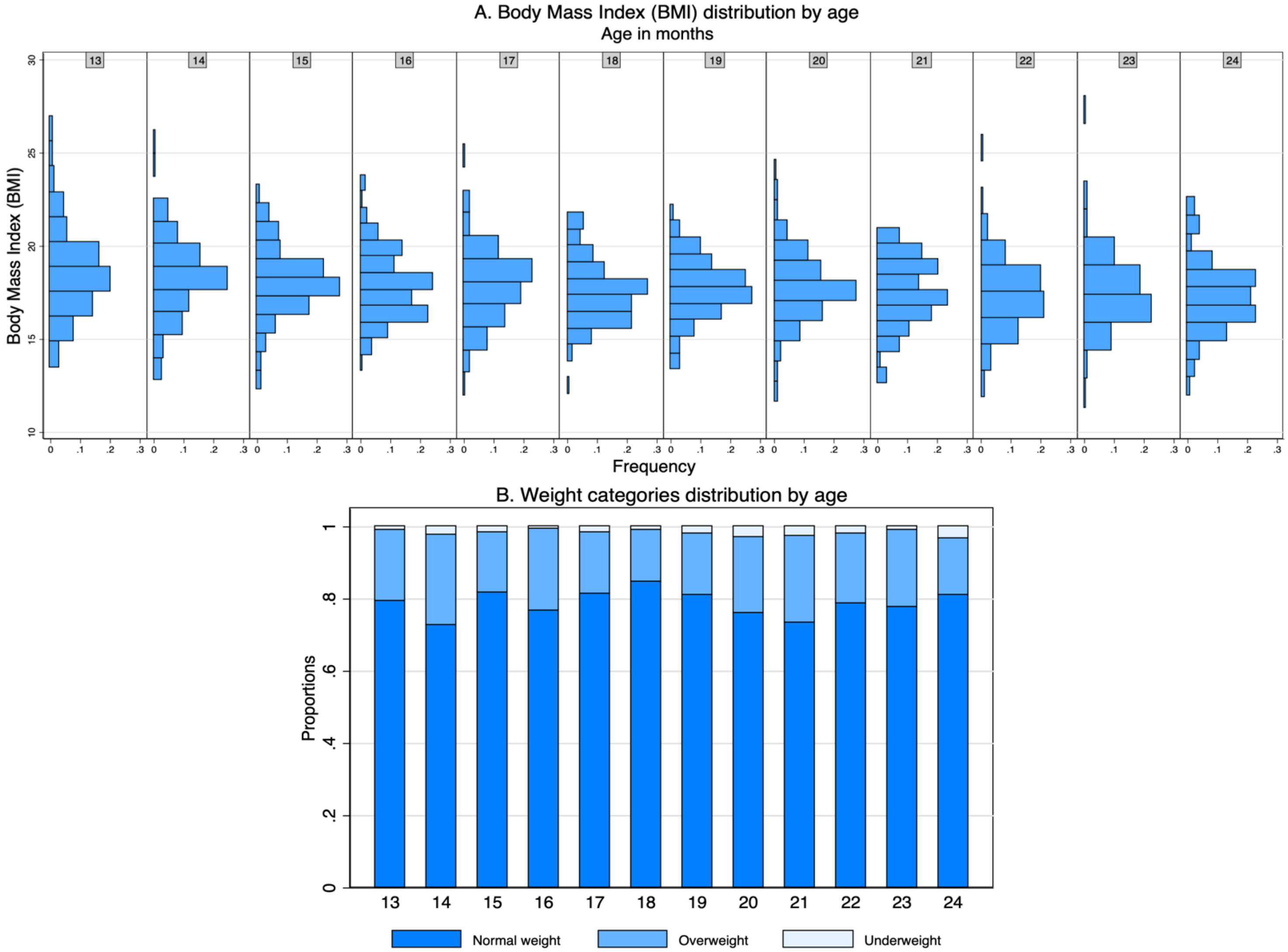


**Notes**: Weights categories are calculated based on Body mass index distributions by age, and categories of nourishment in children aged 12-24 years in 2010.

## 2.2 Propensity score matching estimation and balance of the sample after matching

**Table S1**. Propensity score matching estimation

| Probability of attending center-based care | | |
|---|---|---|
| Main independent outcomes | $\boldsymbol{\beta}$ | **SE** |
| **Child characteristics** | | |
| Age | 0.26 | 0.22 |
| Age squared | -0.01 | 0.01 |
| Female | -0.08 | 0.13 |
| Television | -0.15*** | 0.04 |
| Premature | 0.07 | 0.23 |
| Video-games | -0.01 | 0.06 |
| Sleep | -0.32*** | 0.05 |
| Caesarean | 0.04 | 0.13 |
| Illness | 0.18** | 0.07 |
| Older sibling | -0.18 | 0.16 |
| BMI at birth | 0.20 | 0.14 |
| **Mother characteristics** | | |
| Age | -0.11 | 0.08 |
| Age squared | 0.00 | 0.00 |
| Ethnicity | -0.01 | 0.24 |
| BMI | 0.00 | 0.01 |
| Married | -0.11 | 0.15 |
| Worked before birth | 0.27* | 0.16 |
| Smokes | 0.42* | 0.22 |
| Depression | 0.28 | 0.19 |
| Chronic disease | 0.05 | 0.05 |
| Cigarettes | 0.00 | 0.00 |
| **Household characteristics** | | |
| SES | 0.22** | 0.10 |
| Region | -0.02 | 0.02 |
| **Auxiliary variable** | | |
| Home score | 0.02 | 0.02 |
| Constant | 2.09 | 2.35 |
| Number of observations | 1,273 | |
| $R^2$ | 0.07 | |
| P-Value | 0.00 | |

**Notes:** * p < 0.1; ** p < .05; *** p < .01 (two-tailed tests). BMI: body-mass index; SE: standard error; SES: socioeconomic status.

Figure S2 and Table S2 show the balance of the sample after matching. Figure S2 shows a substantial overlap of individuals by "treatment" group (CBC and maternal care). Table S2 shows PSM created a balanced sample (based on observable characteristics).

**Figure S2.** Propensity score matching overlap, kernel distributions for the probability of attending center-based care.

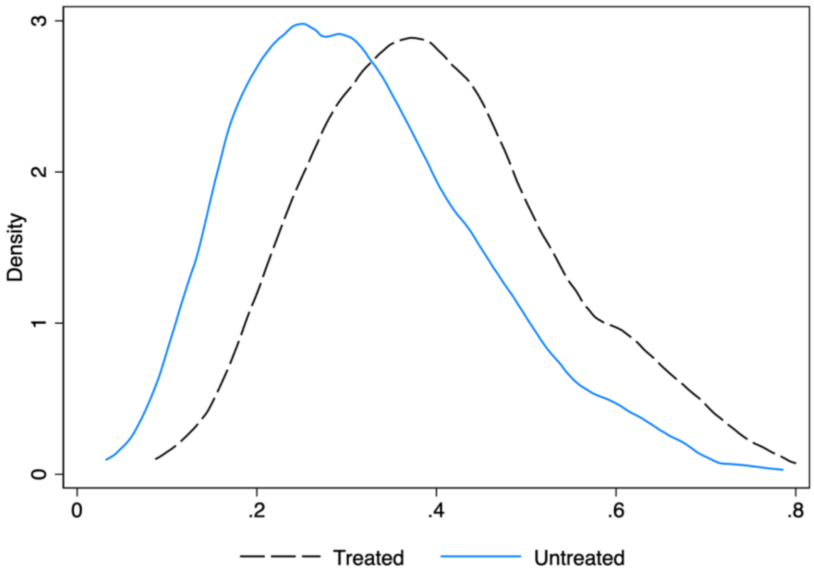


**Notes.** Treated: children attending center-based care at 24-36 months of age.

**Table S2**. Balance of the sample after propensity score matching

| Variable | Matching status | Treated (mean) | Control (mean) | P-value mean difference[a] |
|---|---|---|---|---|
| **Child characteristics** | | | | |
| Age | Unmatched | 18.84 | 18.43 | 0.046 |
| | Matched | 18.84 | 19.15 | 0.172 |
| Female | Unmatched | 0.47 | 0.50 | 0.434 |
| | Matched | 0.47 | 0.51 | 0.281 |
| Television | Unmatched | 2.32 | 2.58 | 0.004 |
| | Matched | 2.32 | 2.32 | 1.000 |
| Premature | Unmatched | 0.08 | 0.08 | 0.594 |
| | Matched | 0.08 | 0.09 | 0.721 |
| Video-games | Unmatched | 0.67 | 0.67 | 0.977 |
| | Matched | 0.67 | 0.67 | 1.000 |
| Sleep | Unmatched | 10.48 | 10.92 | 0.000 |
| | Matched | 10.48 | 10.44 | 0.608 |
| Caesarean | Unmatched | 0.42 | 0.41 | 0.793 |
| | Matched | 0.42 | 0.40 | 0.632 |
| Illness | Unmatched | 0.92 | 0.75 | 0.001 |
| | Matched | 0.92 | 0.92 | 0.970 |
| Older sibling | Unmatched | 0.53 | 0.64 | 0.000 |
| | Matched | 0.53 | 0.54 | 0.840 |
| BMI at birth | Unmatched | 0.34 | 0.28 | 0.029 |
| | Matched | 0.34 | 0.35 | 0.723 |
| **Mother characteristics** | | | | |
| Age | Unmatched | 27.21 | 28.84 | 0.000 |
| | Matched | 27.21 | 27.27 | 0.894 |
| Ethnicity | Unmatched | 0.07 | 0.09 | 0.356 |
| | Matched | 0.07 | 0.07 | 0.895 |
| Overweight status | Unmatched | 57.56 | 64.62 | 0.119 |
| | Matched | 57.56 | 63.90 | 0.110 |
| Married | Unmatched | 0.73 | 0.79 | 0.011 |
| | Matched | 0.73 | 0.71 | 0.548 |
| Worked before birth | Unmatched | 0.22 | 0.19 | 0.231 |
| | Matched | 0.22 | 0.17 | 0.075 |
| Smokes | Unmatched | 0.12 | 0.09 | 0.100 |
| | Matched | 0.12 | 0.12 | 0.916 |
| Depression | Unmatched | 0.15 | 0.11 | 0.037 |
| | Matched | 0.15 | 0.15 | 0.924 |
| Chronic disease | Unmatched | 1.00 | 0.83 | 0.035 |
| | Matched | 1.00 | 0.83 | 0.078 |
| Cigarettes | Unmatched | 6.70 | 6.10 | 0.777 |
| | Matched | 6.70 | 8.22 | 0.553 |
| **Household characteristics** | | | | |
| SES | Unmatched | 0.08 | -0.04 | 0.003 |
| | Matched | 0.08 | 0.12 | 0.396 |
| Region | Unmatched | 8.75 | 8.92 | 0.457 |
| | Matched | 8.75 | 8.89 | 0.603 |
| **Auxiliary variable** | | | | |
| Home score | Unmatched | 15.16 | 14.92 | 0.199 |
| | Matched | 15.16 | 15.14 | 0.906 |
| **Number of observations** | | 1,273 (Matched sample) | | |

**Notes:** "Treated" stands for children who attended center-based care programs. "Control" stands for children in maternal care. Matching status define whether the mean comparison came from unmatched children or matched. BMI: body-mass index; SES: socioeconomic status. [a] P-value was calculated based on mean differences using t-tests between treated and control groups.

## 2.3 Robustness checks: raw results of the association between center-based care and BMI

**Table S3**. Correlation between center-based care and BMI excluding covariates. Estimates are shown for the sample with and without propensity score matching.

| | **OLS DID**[a] | | **GLS FE**[b] | |
|---|---|---|---|---|
| Main Independent Outcomes | ***β*** | **SE** | ***β*** | **SE** |
| **Without propensity score matching** | | | | |
| Center-based care | -0.14 | 0.12 | -0.85*** | 0.09 |
| Constant | -0.71*** | 0.07 | 17.54*** | 0.016 |
| Sample size (n) | 1,273 | | 1,268 | |
| $R^2$ | 0.001 | | 0.048 | |
| P-value (given F-test) | 0.25 (1.29) | | <0.01 (62.44) | |
| **Using propensity score matching** | | | | |
| Center-based care | -0.25** | 0.14 | -0.91*** | 0.12 |
| Constant | -0.69*** | 0.08 | 17.59*** | 0.03 |
| Sample size (n) | 1,273 | | 1,268 | |
| $R^2$ | 0.003 | | 0.077 | |
| P-value (given F-test) | 0.05 (3.40) | | <0.01 (76.78) | |

**Notes:** * p < 0.1; ** p < .05; *** p < .01 (two-tailed tests). DID: difference in differences; FE: fixed-effects; GLS: generalized least squares; OLS: ordinary least squares; PSM: propensity score matching; SE: standard error. Main results from PSM estimation are shown in Table S1 and Table S2.

[a] OLS DID is based on Model 1

[b] GLS FE is based on Model 3

**Table S4**. Results of the full sample analysis including center-based care in comparison with any other type of care

| | OLS DID w/PSM | |
|---|---|---|
| Main Independent Outcomes | ***β*** | **SE** |
| **Child Characteristics** | | |
| Center-based care[a] | -0.23*** | 0.09 |
| Age | 0.26* | 0.15 |
| Age squared | 0.00 | 0.00 |
| Female | 0.27*** | 0.08 |
| Television | 0.01 | 0.03 |
| Premature | -0.04 | 0.17 |
| Video-games | -0.06 | 0.04 |
| Sleep | -0.05 | 0.04 |
| Caesarean | -0.02 | 0.09 |
| Illness | 0.03 | 0.05 |
| Older Sibling | -0.12 | 0.11 |
| BMI at birth | -0.02 | 0.1 |
| **Mother Characteristics** | | |
| Age | 0.06 | -0.05 |
| Age squared | 0.00 | 0.00 |
| Ethnicity | -0.22 | 0.17 |
| BMI | 0.03*** | 0.01 |
| Married | 0.03 | 0.10 |
| Worked before birth | 0.02 | 0.16 |
| Smokes | 0.10 | 0.10 |
| Depression | 0.03 | 0.14 |
| Chronic disease | 0.02 | 0.03 |
| Cigarettes | 0.00 | 0.00 |
| **Household characteristics** | | |
| SES | 0.04 | -0.06 |
| Region | 0.01 | -0.01 |
| Constant | -5.19*** | -1.58 |
| Number of individuals | 2,460 | |
| $R^2$ | 0.04 | |

**Notes:** * p < 0.1; ** p < .05; *** p < .01 (two-tailed tests). BMI: body-mass index; DID: difference-in-differences; OLS: ordinary least squares; PSM: propensity score matching; SE: Standard error; SES: socioeconomic status. PSM was not included.

[a] Stands for center-based care in comparison with any other type of care. Sampled children were aged between 24-48 months in 2012.

Figures S3-S5 show our model diagnostics (Model 1 as reference; OLS DID w/PSM). The figures suggest our model specification is adequate despite a small $R^2$ and meets regression assumptions. Figures S3 (kernel density plot) and S4 (standardized normal and quartile probability plots) show diagnostics for normality of the error terms for model 1 (for valid hypothesis testing), which shows a normally distributed error-term, with an average value ~0 (population mean of zero). Figure S5 shows the residuals versus fitted (predicted) values, which shows homogeneity of variance with no clear sign of heteroscedasticity, which is confirmed by the Breusch-Pagan test for heteroscedasticity (p>0.39).

**Figure S3.** Kernel density plot of the residuals.

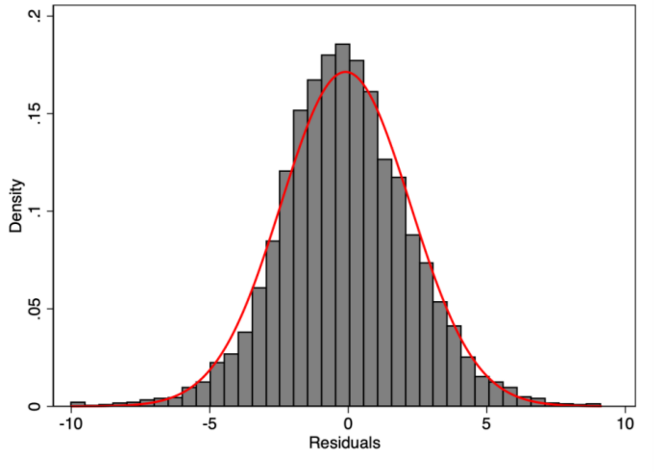


**Figure S4.** Standardized normal and quartile probability plots.

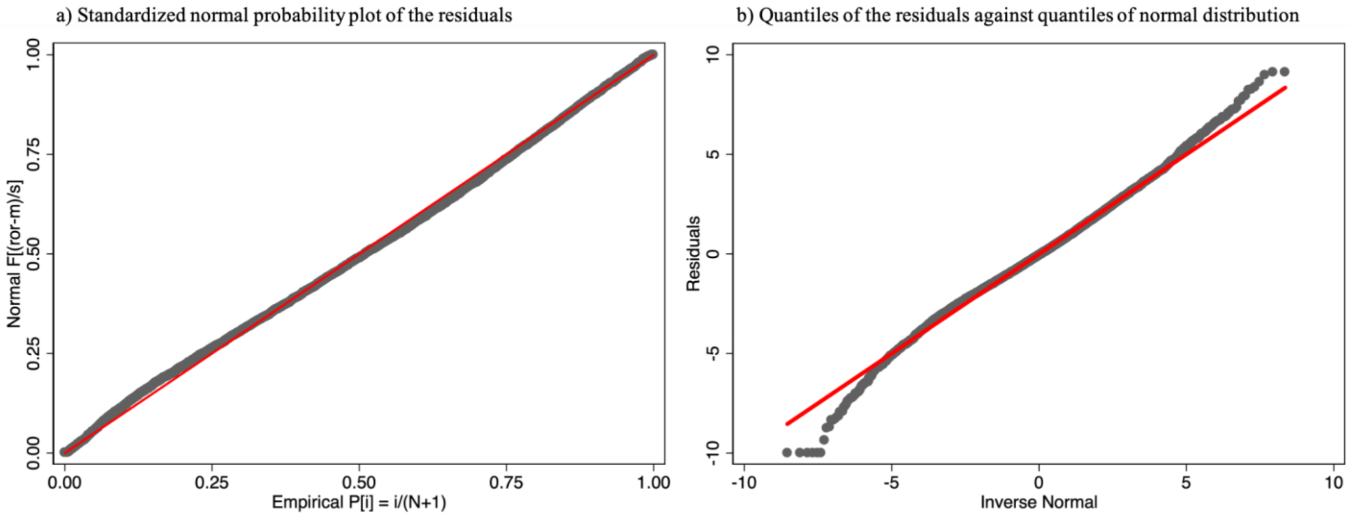

**Figure S5.** Residuals versus fitted predicted values.

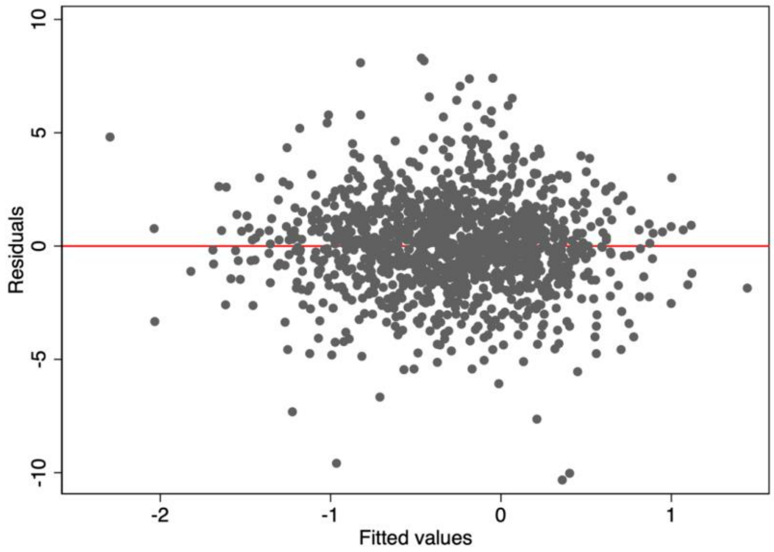

## 2.4 Additional results: mechanisms that could explain main findings

**Table S5**. Lower second and third quintiles of socioeconomic status as moderators of observed changes in body mass index

| | DID w/PSM | | Full DID w/PSM | |
|---|---|---|---|---|
| **Main outcome** | ***β*** | **SE** | ***β*** | **SE** |
| **A1. SES moderators** | | | | |
| **A1.1: SES (40% more vulnerable)** | | | | |
| Center based care | -0.26 | 0.17 | -0.28 | 0.18 |
| SES<40%*Center based care | -0.03 | 0.27 | 0.05 | 0.27 |
| SES<40% | -0.35** | 0.17 | -0.35** | 0.16 |
| Constant | -6.37*** | 2.43 | -0.21 | 0.93 |
| **A1.2: SES (60% more vulnerable)** | | | | |
| Center based care | -0.38* | 0.22 | -0.41* | 0.22 |
| SES<60%*Center based care | 0.16 | 0.27 | 0.23 | 0.28 |
| SES<60% | -0.42** | 0.17 | -0.42** | 0.17 |
| Constant | -6.20** | 2.44 | -0.18 | 0.93 |
| Sample (n) | 1 273 | | 1 268 | |
| $R^2$ | 0.06 | | 0.04 | |
| P-value | 0.00 | | 0.00 | |

**Notes**: * p < 0.1; ** p < .05; *** p < .01 (two-tailed tests). SES: socioeconomic status; BMI: body-mass index; DID: difference-in-differences; PSM: propensity score matching

**Table S6.** Probability of being overweight in 2012 (Model 5)

| | Model 5.1 | |
|---|---|---|
| **Independent outcomes** | **$\beta$ (OR)** | **SE** |
| Center based care | 0.94 | 0.14 |
| **Child characteristics** | | |
| Age | 0.99 | 0.02 |
| Female | 0.72** | 0.10 |
| Television | 1.06 | 0.05 |
| Premature | 0.96 | 0.26 |
| Video-games | 0.85 | 0.15 |
| Sleep | 0.92 | 0.05 |
| Caesarean | 1.41** | 0.21 |
| Older sibling | 0.74* | 0.13 |
| BMI at birth | 0.84 | 0.13 |
| **Mother characteristics** | | |
| Age | 1.01 | 0.01 |
| Ethnicity | 1.12 | 0.29 |
| Overweight | 1.45** | 0.25 |
| Married | 0.96 | 0.17 |
| Smokes | 1.04 | 0.25 |
| Depression | 1.05 | 0.23 |
| Chronic diseases | 1.10** | 0.05 |
| **Household Characteristics** | | |
| SES<80% | 1.56** | 0.36 |
| Region | 1.00 | 0.02 |
| **Auxiliary variable** | | |
| SES*Video-games | 1.00 | 0.20 |
| Constant | 0.47 | 0.55 |
| Number of observations | 1,268 | |
| $R^2$ | 3.11% | |
| P-Value of overall model (F-Test) | <0.001 | |

**Notes**: * p < 0.1; ** p < .05; *** p < .01 (two-tailed tests). *βs* are presented as Odds Ratios (OR). SES: socioeconomic status; BMI: body-mass index. Because playing video games and SES are positively correlated, we added an interaction term to the regression, but found no meaningful results (F-test= 4.3, p-value=.12).
